# Empirically driven definitions of “good,” “moderate,” and “poor” levels of functioning in the treatment of schizophrenia

**DOI:** 10.1007/s11136-012-0335-z

**Published:** 2012-12-14

**Authors:** Haya Ascher-Svanum, Diego Novick, Josep Maria Haro, Jaume Aguado, Zhanglin Cui

**Affiliations:** 1 Lilly Research Laboratories, US Outcomes Research, Eli Lilly and Company, Lilly Corporate Center, Indianapolis, IN 46285 USA; 2 Eli Lilly and Company, Lilly Research Centre, Erl Wood Manor, Sunninghill Road, Windlesham, Surrey, GU20 6PH UK; 3 Sant Joan de Deu-SSM, Fundació Sant Joan de Déu, CIBERSAM, Sant Boi de Llobregat, Barcelona, Spain

**Keywords:** Schizophrenia, Functioning, Quality of life

## Abstract

**Purpose:**

This study used an empirical approach to identify and validate the classification of patients with schizophrenia in “good,” “moderate,” or “poor” functioning groups based on the assessment of functional measures.

**Methods:**

Using data from a study of schizophrenia outpatients, patients were classified into functional groups using cluster analysis based on the Heinrich–Carpenter Quality of Life Scale (QLS), the 36-item Short-Form Health Survey (SF-36) Mental Component Summary Score, and a productivity measure. A three-cluster solution was chosen. Concurrent, convergent, and discriminant validity were assessed. Criteria for classifying patient functioning as “good,” “moderate,” or “poor” were established using classification and regression tree analysis.

**Results:**

The three clusters consistently differentiated patients on the QLS, SF-36 Mental Component Summary Score, and productivity measure. The clusters also differed on other functional measures and were concordant with previous functional classifications. Concurrent, convergent, and discriminant validity were good. “Good” functioning was identified as a QLS total score ≥84.5; “moderate” and “poor” functioning were separated by a cutoff score of 15.5 on the QLS intrapsychic foundation domain. Sensitivity ranged from 86 to 93 % and specificity from 89 to 99 %.

**Conclusions:**

The heterogeneity in functioning of schizophrenia patients can be classified reliably in an empirical manner using specific cutoff scores on commonly used functional measures.

## Introduction

Schizophrenia is often a severe and persistent mental illness typically accompanied by functional impairment and disability [1], characterized by poor psychosocial functioning, difficulties in activities of daily living and interpersonal relationships, low levels of productivity, and high rates of unemployment [2–4]. There is marked heterogeneity in the level of psychosocial functioning among patients with schizophrenia; some patients can function with mild difficulties, while others are severely impaired and unable to live independently.

A number of instruments have been developed in the field of schizophrenia research to assess the patients’ health-related quality of life to help clinicians and researchers better assess patients’ health and function. However, lack of a gold standard with which to compare instrument-based scores has limited their usefulness [5]. At present, the health-related quality of life instrument used most frequently in schizophrenia research is the Heinrich–Carpenter Quality of Life in Schizophrenia Scale (QLS) [6], a clinician-rated scale of patients’ social, occupational, and psychological functioning. Interestingly, despite the extensive use of the QLS in schizophrenia research, the interpretation of QLS scores has rarely been studied. While higher scores indicate better functioning of schizophrenia patients, there are no studies empirically delineating specific cutoff scores that correspond to various levels of functioning. The QLS is not alone, as the interpretability of other health status assessment tools in the treatment of schizophrenia has rarely been a topic of investigation. To our knowledge, the only previous attempt to empirically interpret scores of a health status measure in schizophrenia was made by Cramer et al. [7] who studied the QLS and identified that “improved” status corresponds to 26 percent increase in QLS scores and “much better” status is associated with a 50 percent increase in QLS scores. That study determined the average magnitude of change in QLS scores that is associated with clinician-detected improvement or deterioration, thus clarifying the meaning of a clinically detectable improvement, but it does not enable clinicians and schizophrenia researchers to identify what a specific QLS score may mean, because a patient may be “much improved” but still exhibit a relatively poor level of functioning. At present, it is unclear which QLS scores reflect a patient’s level of functioning as “good,” “moderate,” or “poor,” and no cutoff scores have been yet delineated to identify each functional level category. A few previous attempts have been made to classify schizophrenia patient functioning according to severity level [8, 9], but none have used an empirically driven approach that focused only on functioning. While Lipkovich et al. [8] created a data-driven classification that combined symptomatology and functioning using the QLS and the Positive and Negative Syndrome Scale [10], Stahl et al. [9] used theoretically based criteria to classify patients using the QLS. Previous research has shown that there remains a need for a data-driven classification based on measures of functioning. Classification of schizophrenia patients into distinct levels of functioning may be useful for translating absolute scale scores into meaningful and relevant categories, facilitating interpretation of the scores in clinical practice and schizophrenia research. Thus, the identification of categories may facilitate comparison among studies and the translation of the evaluation of the patient into terms that can be easily communicated to the patients and their families. The primary objective of this analysis was to identify, using an empirical approach, the equivalence of “good,” “moderate,” and “poor” levels of functioning on various functional measures. A secondary objective was to assess the construct validity of the new functional category scores in the treatment of outpatients with schizophrenia. We hypothesized that compared to patients with “poor” or “moderate” levels of functioning on the QLS, those with “good” functional levels will exhibit lower schizophrenia symptom severity levels, greater levels of productive activity, and higher scores on other health-related quality of life (QOL) measures, including patient self-reported scales like the SF-36 and EQ-5D and clinician-rated scales like the schizophrenia objective functioning instrument (SOFI). We also hypothesized that differentiation between patients’ levels of functioning on the QLS will be best accomplished not only by the total score on the scale, but also that patients’ drive, sense of purpose, and motivation (measured in the QLS by the intrapsychic domain) will play an important role, more so than other domains, as patients’ drive and initiative were found to be robust predictors of overall QOL, as measured by QLS, than actual accomplishments and satisfaction [11].

## Patients and methods

### Study design and patient population

This analysis used baseline data from a 2-year multicenter, randomized, open-label study comparing the long-term treatment effectiveness and safety of olanzapine long-acting injection with oral olanzapine (HGLQ, ClinicalTrials.gov registration number NCT 00320489) [12]. Ethics approval for the study was granted by the research committees of the participating centers following country regulations. All participants gave their informed consent prior to inclusion. The study included 524 outpatients who met diagnostic criteria for schizophrenia and were considered to be at risk of relapse. Patients with DSM-IV- or DSM-IV-TR-defined substance (except nicotine and caffeine) dependence within the past 30 days were excluded from the study. The study design consisted of two periods: a screening phase during which the patients were screened for eligibility (visits 1–2) and an open-label treatment phase lasting up to 104 weeks. Patients were treated with oral or long-acting injection during the course of the study. Other antipsychotics, mood stabilizers, and anticonvulsants were not allowed. Anticholinergics, antidepressants, except fluvoxamine, which were started before study initiation, and several benzodiazepines (up to a dose equivalent to diazepam 30 mg/day) were allowed.

### Measures

Patients’ levels of functioning were assessed using the following 5 measures: the Quality of Life Scale (QLS), the Medical Outcomes Study Short Form-36 (SF-36), the SOFI, the EuroQol-5 Dimensions Questionnaire (EQ-5D), and patient productivity level.

The QLS [6] is an interviewer-rated scale used to assess the health-related level of functioning in patients with schizophrenia and includes while balancing subjective questions regarding life satisfaction and objective indicators of social and occupational role functioning. The QLS is widely used in clinical trials of antipsychotic medications and in course of illness studies [13]. The QLS consists of 21 items that are assessed during a semi-structured interview. Completion requires approximately 45 min, during which various topics are explored using specified probes. Each item is rated on a 7-point (0–6) scale. High numbers reflect normal or unimpaired functioning, and low scores reflect severely impaired functioning. The range of possible total score is 0–126 points. The scale contains 4 subdomains: intrapsychic foundations (e.g., degree of motivation, scored from 0 = “Lack of motivation significantly interferes with basic routine” to 6 = “No evidence of significant lack of motivation”), interpersonal relations (e.g., level of social activity, scored from 0 = “virtually absent” to 6 = “Adequate level of regular social activity”), instrumental role (e.g., extent of occupational role functioning, scored from 0=“virtually no role functioning” to 6=“full time or more”), and common objects and activities (e.g., time utilization, scored from 0 = “Spends the vast majority of his/her day in aimless inactivity” to 6 = “No excessive aimless inactivity beyond the normal amount required for relaxation”). The QLS has been shown to have acceptable psychometric qualities: Test–retest reliability is good for nearly all items of the scale, categories, and overall score. Internal consistency alpha coefficients were 0.8–0.9 for the global score, and convergent validity is good [14–16]. Cramer et al. [17] reported that the QLS appeared to be substantially more sensitive to subtle change and treatment effects than a patient-reported QOL measure for clinical trials. Although the QLS has been used extensively in schizophrenia research, interpretation of the scale score has never been clarified beyond stating that higher scores mean better functioning. Due to lack of scale cutoff scores for various levels of functioning, it is currently unclear which scores may reflect patients’ “good,” “moderate,” or “poor” levels of functioning.

The Medical Outcomes Study Short Form-36 (SF-36) [18] is a patient-rated health status measure, one of the most widely used QOL evaluation tool in the world to date. It consists of 36 questions covering 8 areas of functioning and well-being (physical function, bodily pain, role limitations due to physical problems, vitality, general health perceptions, role limitations due to emotional problems, mental health, and social function). Each scale is linearly transformed into a 0-100 scale with higher scores representing better health status and functioning. In addition to scores for the 8 areas, there are 2 component scores, the Mental Component Summary Score and Physical Component Summary Score, in which the standardized scores have a mean of 50 with a standard deviation of 10. These component summary measures have features that make them more advantageous for use in clinical trials, including higher measurement precision, reduced floor and ceiling effects, simpler analytic outcomes, and superior responsiveness [19]. The reliability and validity of the SF-36 in the treatment of patients with schizophrenia has been previously studied, showing the SF-36 can be a reliable and valid measure of perceived functioning and well-being for schizophrenia patients [20]. In the current study, we assessed patients’ mental health functioning using the Mental Component Summary Score of the SF-36.

The SOFI [21] was developed to measure community functioning and has four domains: (1) living situation, (2) instrumental activities of daily living, (3) productive activities, and (4) social functioning. Items from these domains were scored by the participating investigators and were combined to provide a global score, with higher scores indicating a better level of functioning. The psychometric properties of the SOFI have been studied [21], showing good evidence supporting reliability and construct validity. For example, the values for test–retest reliability were >0.70, inter-rater reliability ICCs ranged from 0.50 to 0.79, and the SOFI demonstrated adequate construct validity based on correlations with other QOL measures like the QLS. Discriminant validity was also supported based on SOFI score comparisons between patient groups identified using PANSS scores.

The EuroQol-5 Dimensions Questionnaire (EQ-5D) general tariff and visual analogue scale (EQ-5D VAS). The EQ-5D is a generic questionnaire generating a health profile and a single index score for health-related QOL. The general tariff of the EQ-5D [22, 23] uses population norms to transform a patient’s mean scores on the scale’s 5 items (mobility, self-care, usual activities, pain/discomfort, and anxiety/depression) into a single rating ranging from 0 (death) to 1 (best). The EQ-5D tariffs have been shown to be stable across different European countries [24]. Furthermore, patients self-rated their current health status using the EQ-VAS, on a scale that ranged from 0 (worst imaginable health) to 100 (best imaginable health). The validity of the EQ-5D in assessing and valuing health status in patients with schizophrenia has been shown to be reasonable, despite a moderate ceiling effect [25].

Patient productivity level [26] was evaluated by the participating investigator as measured by the percentage of time the patient was involved in functional activities or work (including work for pay, being a student, housekeeping, and volunteer work) in the 3 months prior to enrollment. This was assessed as a single item rated on a 5-point scale: (1) no useful functioning; (2) functional activities occupied >0–25 % of the time; (3) functional activities occupied >25–50 % of the time; (4) functional activities occupied >50–75 % of the time; and (5) functional activities occupied >75–100 % of the time. The psychometric properties of this brief measure have not been previously studied. Higher scores on this measure were found [26] to be significantly associated with higher study completion rates and better scores on the Positive and Negative Symptom Scale (PANSS).

In addition to the measures of functioning, the patient illness severity level was assessed with the Positive and Negative Syndrome Scale, the PANSS [27], which is the most widely used measure of symptom severity level in schizophrenia research. The PANSS has 30 items, which are rated on a scale from 1 (absent) to 7(extreme). The PANSS scores are typically presented for the total scale and separately for positive symptoms, negative symptoms, and general psychopathology. Positive symptoms include delusions, hallucinatory behavior, and suspicion/persecution, whereas negative symptoms include blunted affect, emotional withdrawal, poor rapport, and passive/apathetic social withdrawal. Symptoms of general psychopathology include conceptual disorganization, disorientation, poor attention, excitement, hostility, poor impulse control, anxiety, and depression. The meaning of the PANSS total scores has been previously delineated in an empirical manner [10] where a total score of 58 corresponds to being “mildly ill,” a score of 75 to being “moderately ill,” a score of 95 to “markedly ill,” and a PANSS score of 116 to “severely ill.” The psychometric properties of the PANSS are currently well documented [28–30] showing good validity and reliability. More recent studies have shown the PANSS to have sound construct validity [31, 32], external validity [33], and good internal consistency of its five-factor structure, with Cronbach’s alpha >0.70 [31, 33].

### Statistical analysis

#### Cluster definitions

Consistent with prior research [8], a hierarchical cluster analysis based on Ward’s minimum-variance method was used to define groups of patients using baseline values for the QLS total score and the four QLS subscale scores, productivity levels, and SF-36 Mental Component Summary Score. These variables were chosen since they evaluate functioning and QOL. The clustering procedure was applied to standardized data. The number of clusters was chosen based on the proportion of variation in the data (*R* ^2^) captured by the clusters; the decision was determined when a reduction in the number of groups led to a substantial deterioration in *R* ^2^. Baseline socio-demographics and clinical characteristics were described for each cluster.

#### Cluster validation

The construct validity of the three clusters (“good,” “moderate,” and “poor”) was assessed using concurrent, convergent, and divergent validity. Concurrent validity is customarily assessed by comparing the results of a new measure with results of a “gold standard” obtained at approximately the same point in time, so that both measures reflect the same construct. Considering the absence of a “gold standard,” this analysis assessed concurrent validity by comparing the clusters with a theoretically driven definition based on the QLS [9]. This approach involved comparing the proportion of patients deemed to have (as per Stahl et al.) “adequate psychosocial functioning” in each of the newly identified functional level clusters, where adequate psychosocial functioning for each QLS subscale was defined as a baseline score ≥4 (“at least some consistent functioning”) on all items within that QLS subscale [9]. Another approach compared the clusters with a previous, empirically driven criteria [8], which defined “good functioning” using scores on the QLS instrumental and interpersonal domains and the productivity measure.

Convergent validity is typically determined by examining the overlap between two or more measures that are presumed to assess the same construct. In this analysis, convergent validity was assessed by comparing the functional clusters using the EQ-5D scale (EQ-5D tariffs; EQ-5D VAS scores; EQ-5D VAS > 70) [34] and the SOFI global scale scores.

Divergent validity examines the extent to which a measure correlates with attributes that are different from the attribute the measure is intended to assess; that is, whether measures that should not be related are not. In this analysis, divergent validity was assessed by looking for associations between each of the functional clusters and two parameters that are generally not expected to be related to level of functioning: age and gender.

#### Analysis of differences between clusters

Statistical differences in variables between clusters were assessed using analysis of variance for continuous variables and chi-square tests for categorical variables

#### Classification and regression tree analysis

Classification and regression tree (CART) analysis allows the classification of patients into distinct groups based on dichotomous criteria and was employed to define the rules used to classify patients in the clusters [35]. The sensitivity and specificity of the CART classification were calculated to assess goodness of fit of the CART analysis.

## Results

### Baseline characteristics of the study sample

Baseline characteristics of the study sample are shown in Table 1. More than two-thirds of the patients were male, and the mean age was 40.9 years. The mean PANSS total score was 56.7, indicating that patients were mildly ill [10]. Productivity levels ranged from 0 % (no time involved in functional activities) to 100 % of time involved in functional activities, with the bulk of patients (roughly 60 %) spending up to 50 % of their time involved in productive activities.

**Table 1:** Baseline characteristics of the study sample

Characteristic	
Male gender, *n* (%)	352 (67.2)
Age, years	40.9 (10.9)
Age at illness onset, years	26.2 (8.9)
Illness duration, years	14.8 (10.5)
PANSS total score	56.7 (9.3)
Productivity level: amount of functional activities/work, *n* (%)	
0	74 (14.2)
>0–25	189 (36.3)
>25–50	129 (24.8)
>50–75	98 (18.8)
>75–100	31 (5.9)
QLS total score	63.1 (20.1)
QLS common objects and activities	6.6 (2.6)
QLS intrapsychic foundation	21.8 (7.4)
QLS interpersonal relations	20.5 (9.6)
QLS instrumental role	14 (3.4)
SOFI global score	60.6 (18.4)
EQ-5D VAS score	66.9 (23.2)
EQ-5D general tariff score (1–5)	0.7 (0.3)
SF-36 Mental Component Summary Score mental composite score	41.2 (11.5)

Data are presented as mean (SD) unless indicated otherwise

*EQ*-*5D* EuroQol-5 Dimensions Questionnaire, *PANSS* Positive and Negative Symptom Scale, *QLS* Quality of Life Scale, *SF*-*36* Medical Outcomes Study Short Form-36, *SOFI* Schizophrenia Objective Functioning Scale, *SD* standard deviation, *VAS* visual analogue scale

### Cluster selection

**Fig. 1:**
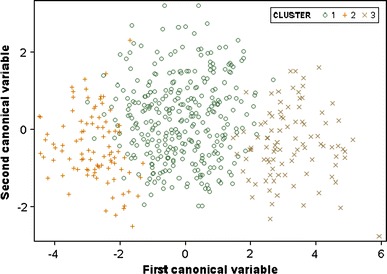
Graphical presentation of the three-cluster solution: cluster 1 = “moderate” functioning, cluster 2 = “poor” functioning, and cluster 3 = “good” functioning. [For color reproduction]

Based on the proportion of variation in the data (*R* ^2^) captured by the clusters, and determined when a reduction in the number of groups led to a substantial deterioration in *R* ^2^, the number of clusters was chosen as three. Estimated *R* ^2^ for the two-cluster solution was 0.311, compared with 0.457 for the three-cluster solution, and 0.508 for four clusters. The three-cluster solution was chosen to maximize simplicity, explanatory power, and separation between the groups. Figure 1 is a graphical representation of the three-cluster solution; the bulk of the patients appears in cluster 2, flanked by smaller but roughly equal numbers of patients in cluster 3 and cluster 1.

### Cluster description

Baseline functional measure scores for patients in each of the functioning clusters are shown in Table 2. As expected, functioning (assessed using QLS total scores and scores from the four QLS subscales) was highest in the good cluster and deteriorated from “good” to “moderate” to “poor.” A similar pattern of deterioration from “good” through “moderate” to “poor” was seen for the SF-36 Mental Component Summary Score (indicating general mental health status) and productivity levels. The bulk of the patients were in the “moderate” functioning cluster, with fewer (roughly equal numbers of) patients in the “good” and “poor” clusters.

**Table 2:** Baseline functional measure scores by functioning cluster

Functional measure	“Good”	“Moderate”	“Poor”
QLS total score	93.5 (8.8)	61.9 (10.7)	37.3 (7.6)
QLS common objects and activities	9.4 (1.5)	6.6 (1.9)	3.6 (1.6)
QLS intrapsychic foundation	31.7 (3.6)	21.8 (4.5)	11.9 (3.5)
QLS interpersonal relations	34.0 (6.2)	19.7 (6.4)	10.1 (4.8)
QLS instrumental role	18.2 (2.8)	13.5 (2.8)	11.7 (2.2)
SF-36 Mental Component Summary Score	44.4 (10.0)	41.1 (11.5)	38.4 (12.4)
Productivity level	3.5 (1.2)	2.7 (1.0)	1.6 (0.6)
Number of patients	95	321	96

Data are presented as mean (SD)

*QLS* Quality of Life Scale, *SF*-*36* Medical Outcomes Study Short Form-36, *SD* standard deviation

Baseline patient characteristics by level of functioning are shown in Table 3. The percentage of male patients, mean age, age at illness onset, and illness duration appeared to be roughly similar across the clusters. Symptom severity according to PANSS total score, negative and general psychopathology subscale scores (*P* < 0.0001) and PANSS positive subscale score (*P* < 0.01) was significantly different across the three clusters; symptom severity was best (lowest) in the “good” functioning cluster and worst in the “poor” functioning cluster. Productivity levels were significantly different across the three clusters (*P* < 0.0001) and were highest in the “good” functioning cluster and lowest in the “poor” functioning cluster; 25.3 % of patients in the “good” functioning cluster and 0 % of patients in the “poor” functioning cluster had >75–100 functional activities in the last 3 months, and 3.2 % of patients in the “good” functioning cluster and 42.7 % of patients in the “poor” functioning cluster had no functional activities in the 3 months prior to enrollment. There were no differences in distribution of patients in each of the clusters for each treatment group.

**Table 3:** Baseline patient characteristics by level of functioning

Parameter	“Good”	“Moderate”	“Poor”	*P* value for pairwise comparisons		
				“Good” versus “moderate”	“Good” versus “poor”	“Moderate” versus “poor”
Number of patients	95	321	96			
Male gender, *n* (%)	60 (63.2)	218 (67.9)	66 (68.8)	0.3872	0.4148	0.8773
Age, years	39.2 (10.9)	41.1 (10.9)	42.0 (10.6)	0.1895	0.0745	0.3499
Age at illness onset, years	26.1 (8.5)	26.1 (8.9)	27.1 (9.4)	0.9533	0.5170	0.4509
Illness duration, years	13.1 (10.4)	15.0 (10.4)	14.9 (10.1)	0.0683	0.1257	0.9019
PANSS total score*	51.1 (10.6)	57.1 (8.4)	59.9 (8.3)	<0.0001	<0.0001	0.0014
PANSS negative subscale score*	14.2 (3.9)	15.9 (4.0)	17.8 (3.7)	0.0018	<0.0001	<0.0001
PANSS positive subscale score**	11.7 (3.7)	12.9 (3.4)	12.8 (3.5)	0.0022	0.0270	0.7090
PANSS general psychopathology score*	25.2 (5.6)	28.3 (4.8)	29.3 (5.3)	<0.0001	<0.0001	0.1086
Proportion of patients in each productivity level, %*						
0	3.2	9	42.7	<0.0001	<0.0001	<0.0001
>0–25	22.1	34.6	54.2			
>25–50	17.9	33.0	3.1			
>50–75	31.6	21.2	0.0			
>75–100	25.3	2.2	0.0			

Data are presented as mean (SD) unless indicated otherwise

*SD* standard deviation, *PANSS* Positive and Negative Symptom Scale

Significant differences across the three levels of functioning: * *P* < 0.0001; ** *P* < 0.01

Pairwise comparisons revealed that there were significant differences between the “good” and “moderate” clusters, and between the “good” and “poor” clusters in PANSS total, negative, positive, and general psychopathology subscale scores, and in the proportion of patients with no functional activities (Table 3). There were significant differences between the “moderate” and “poor” clusters only in PANSS total and negative subscale scores and in the proportion of patients with no functional activities.

### Cluster validation

#### Concurrent validity

Concurrent validity (i.e., the proportion of patients with “adequate psychosocial functioning”) across the three clusters was “good”; the proportion of patients with adequate psychosocial functioning in all QLS subscales was greatest in the “good” functioning cluster, intermediate in the “moderate” functioning cluster, and lowest in the “poor” functioning cluster (Table 4) (*P* < 0.0001 for differences across the three levels of functioning). For example, 60.0 % of patients in the “good” functioning cluster fulfilled the criteria for adequate intrapsychic foundation, while only 2.5 % of patients in the “moderate” functioning cluster and 0 % of patients in the “poor” functioning cluster fulfilled this criterion.

**Table 4:** Concurrent validity; the proportion of patients in the “good,” “moderate,” and “poor” functional clusters with an adequate level of functioning for each QLS subscale and according to the empirical definition of functioning

Measure	“Good”	“Moderate”	“Poor”	*P* value for pairwise comparisons		
				“Good” versus “moderate”	“Good” versus “poor”	“Moderate” versus “poor”
QLS subscale (Stahl et al. [7])						
Adequate intrapsychic foundation*	57 (60.0)	8 (2.5)	0 (0.0)	<0.0001	<0.0001	0.2070
Adequate interpersonal relations*	34 (35.8)	2 (0.6)	0 (0.0)	<0.0001	<0.0001	1.0000
Adequate instrumental role*	49 (51.6)	17 (5.3)	0 (0.0)	<0.0001	<0.0001	0.0166
Adequate common objects/activities*	87 (91.6)	108 (33.6)	2 (2.1)	<0.0001	<0.0001	<0.0001
Empirically defined criteria (Lipkovich et al. [6])*	91 (95.8)	196 (61.1)	31 (32.3)	<0.0001	<0.0001	<0.0001

Data are presented as number (%)

*QLS* Quality of Life Scale

Significant differences across the three levels of functioning: * *P* < 0.0001

Based on the second approach (i.e., the proportion of patients meeting empirically defined criteria that included functioning and symptom severity), pairwise comparisons revealed significant differences between the “good” and “moderate” clusters, and between the “good” and “poor” clusters in the four QLS subscales and the empirically defined criteria by Lipkovich et al. [8] (Table 4). There were significant differences between the “moderate” and “poor” clusters only in the QLS subscales of instrumental role and common objects and activities, and empirically defined criteria.

#### Convergent validity

Convergent validity (assessed by comparing the clusters using the EQ-5D scale and SOFI scale scores) was “good”; mean SOFI global scores and EQ-5D general score tariffs were highest in the “good” functioning cluster, intermediate in the “moderate” functioning cluster, and lowest in the “poor” functioning cluster (Table 5) (*P* < 0.0001 for differences across the three levels of functioning). Mean EQ-5D VAS scores and the percentage of patients with EQ-VAS scores >70 were also highest in the “good” functioning cluster, intermediate in the “moderate” functioning cluster, and lowest in the “poor” functioning cluster (*P* < 0.05 for differences across the three levels of functioning).

**Table 5:** Convergent validity; comparisons between the functional clusters using measures that are presumed to assess the same construct—the SOFI and the EQ-5D scale

Measure	“Good”	“Moderate”	“Poor”	*P* value for pairwise comparisons		
				“Good” versus “moderate”	“Good” versus “poor”	“Moderate” versus “poor”
SOFI global score*	78.2 (12.9)	61.7 (14.3)	39.7 (14.6)	<0.0001	<0.0001	<0.0001
EQ-5D general score tariff*	0.84 (0.16)	0.72 (0.27)	0.61 (0.37)	0.0002	<0.0001	0.0131
EQ-5D VAS**	71.4 (21.1)	66.0 (22.1)	64.2 (28.2)	0.0128	0.0977	0.6992
EQ-5D VAS score >70**	54.3	39.7	38.3	0.0121	0.0282	0.8084

Data are presented as mean (SD) or %

*SOFI* Schizophrenia Objective Functioning Scale, *EQ*-*5D* EuroQol-5 Dimensions Questionnaire, *VAS* visual analogue scale

Significant differences across the three levels of functioning: * *P* < 0.0001; ** *P* < 0.05

Pairwise comparisons revealed that there were significant differences between the “good” and “moderate” clusters in all measures (SOFI global score, EQ-5D general score tariff, EQ-5D VAS, and EQ-5D VAS score >70) (Table 5). There were significant differences between the “good” and “poor” clusters in SOFI global score, EQ-5D general score tariff, and the EQ-5D VAS score >70, but not the EQ-5D VAS. There were significant differences between the “moderate” and “poor” clusters only in SOFI global score and EQ-5D general score tariff.

#### Divergent validity

The divergent validity of the clusters (assessed by looking for associations between the clusters and parameters that should not be related) was “good,” as shown by the lack of significant differences between the clusters on age and gender (Table 3).

#### Classification and regression tree analysis

**Fig. 2:**
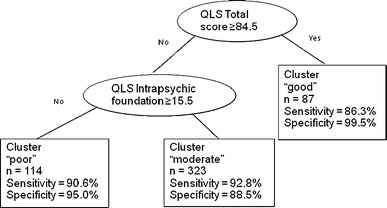
Classification and regression tree (CART) analysis

CART analysis defined cutoff points to classify the patients into the three clusters. Patients with a QLS total score >84.5 were classified as having “good” functioning, whereas “moderate” and “poor” functioning were separated by a cutoff score of 15.5 on the QLS intrapsychic foundation domain (Fig. 2). Using this definition of clusters, compared with the empirically defined criteria, sensitivity ranged from 86 to 93 % and specificity ranged from 89 to 99 %.

## Discussion

This study presents an empirical classification of patients with schizophrenia based on their level of functioning that helps us understand what given scores on a commonly used functional measure (the QLS) mean in clinical practice, and thus aids the translation of research findings into clinical practice. Improving a patient’s level of functioning is an important aim of the treatment, as symptom improvement is no longer considered a sufficient goal in the long-term treatment of patients with schizophrenia [36]. “Poor” levels of functioning were previously found to be related to patient QOL [37] and to treatment cost [38] and thus are of importance not only to patients and their family members, but also to mental health providers, health care decision makers and payers.

To our knowledge, this is the first study to empirically delineate the meaning of functional measure scores in the treatment of patients with schizophrenia. Although one prior study used a theoretically or consensus-based classification of patient functioning [9], and another study [8] used an empirically driven classification incorporating symptoms and functioning, to our knowledge, no previous study has used an empirically driven classification of functional measures alone. For the validation of the new clusters, and in the absence of a “gold standard,” we used prior definitions of functional levels from these two previous studies to assess the construct validity of the new classification of “good,” “moderate,” and “poor” levels of functioning.

Consistent with our hypothesis, patients with a “good” level of functioning had a significantly lower severity level of schizophrenia symptoms, greater levels of productive activity, and higher scores on other health-related QOL measures. This differentiation was consistently observed when using patient self-reported measures (SF-36 and EQ-5D) or a clinician-rated scale (SOFI), demonstrating the robustness and the utility of patient-reported health-related outcomes even when reported by persons diagnosed with and treated for a psychotic disorder like schizophrenia. Our finding of a significant link between a patient’s level of functioning and their level of illness severity per PANSS scores is also consistent with prior research [39–42]. This link was found despite the fact that the study participants were only mildly ill, and differentiation on illness severity levels among mildly ill patients is typically difficult (the “floor effect”). Nonetheless, the new classification of levels of functioning was sufficiently sensitive to detect minor gradations of PANSS scores in the mildly ill range, with a mean PANSS total score of 51, 57, and 60 linked to “good,” “moderate,” and “poor” levels of functioning, respectively.

Current findings have also supported another study hypothesis that differentiation between patients’ levels of functioning on the QLS will be best accomplished not only by the total score on the scale, but by also incorporating patients’ drive, sense of purpose and motivation. This was demonstrated in the CART analysis, which identified cutoff points to classify patients into the three clusters and found—with a high level of sensitivity and specificity (sensitivity ranged from 86 to 93 % and specificity ranged from 89 to 99 %)—that differentiation was maximized when using the QLS total score and the QLS intrapsychic foundation domain, which measures patients’ drive and motivation. Thus, when using the QLS, patients with a total score >84.5 were classified as having “good” functioning, whereas “moderate” and “poor” functioning were separated by a cutoff score of 15.5 on the QLS intrapsychic foundation domain. Moreover, in the process of validating the new clusters (convergent validity), this study has also identified what “good,” “moderate,” and “poor” levels of functioning (per QLS) corresponded to on two other functional measures: a generic measure (EQ-5D) and a disease-specific measure (SOFI), demonstrating that the current findings may be of utility beyond the QLS, in clinical or research settings where these other health-related QOL measures are being used. Overall, the construct validation of the three functional levels (per concurrent, convergent, and divergent validity) suggests that the resulting classification is valid, showing consistent and statistically significant differentiation between the clusters in the expected direction.

This study found that approximately 60 % of the patients were classified as having a “moderate” level of functioning, while a smaller proportion was classified as having either a “good” or a “poor” level of functioning (20 % each). This distribution of functional clusters should not be generalized to other patients with schizophrenia, as it is specific to the patient population enrolled in the study. One should expect heterogeneity among schizophrenia patients on their levels of functioning, and these levels are likely to shift over time, across phases of the illness, and differ between patients observed in usual clinical settings as compared to controlled clinical trials.

## Limitations

Several limitations need to be considered when interpreting the results of this analysis. Firstly, study participants were only mildly ill; thus, it is unclear whether the current findings can be generalized to patients with more severe levels of symptoms. Secondly, as this was a cross-sectional study, the predictive validity of the functional definitions was not assessed. Third, divergent validity was only assessed by comparing gender, age, and age of onset of the three clusters, but comparison has been made with a scale measuring a different constructs. Finally, as this analysis was conducted in a patient population participating in a randomized clinical trial, the classification of functioning will require replication in schizophrenia patients treated in the usual care setting.

## Conclusion

The substantial heterogeneity in levels of functioning among schizophrenia patients can be reliably classified in an empirical manner using specific cutoff scores on a commonly used functional measure, the QLS. Validity assessment of the classes and classification method has been shown to be “good.” While further research is needed to replicate these results, the current findings have utility in the translation of assessment scales into relevant clinical categories.

## Abbreviations

CART: Classification and regression tree
EQ-5D: EuroQol-5 Dimensions Questionnaire
PANSS: Positive and Negative Symptom Scale
QLS: Quality of Life Scale
SF-36: Medical Outcomes Study Short Form-36
SOFI: Schizophrenia Objective Functioning Scale
SD: Standard deviation
VAS: Visual analogue scale
